# Hepatopulmonary Syndrome Diagnosis in the ICU: The Relevance of Bedside Contrast Saline Echocardiography

**DOI:** 10.7759/cureus.52658

**Published:** 2024-01-21

**Authors:** Rita Morais Passos, Francisca Cardoso, Francisco Teixeira da Silva, Rogério Corga da Silva, José Caldeiro

**Affiliations:** 1 Critical Care Department, Hospital de Santa Luzia, Viana do Castelo, PRT; 2 Escola de Medicina, Universidade do Minho, Braga, PRT

**Keywords:** orthodeoxia, saline contrast echocardiography, pulmonary vascular dilations, chronic liver disease, hepatopulmonary syndrome

## Abstract

Hepatopulmonary syndrome (HPS) is an underdiagnosed complication of chronic liver disease (CLD) characterised by the presence of hypoxaemia due to intrapulmonary vascular dilatations. We present two cases of HPS diagnosed during their stay in the ICU. Both patients had a medical history of alcoholic CLD with portal hypertension (PH). The first patient was transferred to the ICU for acute hypoxic respiratory failure (AHRF) due to decompensated cirrhosis with large-volume hydrothorax and diagnosis of acute-on-chronic liver failure (ACLF) grade 2. The presence of orthodeoxia, an alveolar-arterial oxygen gradient (O_2_ A-a grad) of 27 mmHg and positive contrast saline echocardiography confirmed the HPS diagnosis. The second patient was transferred to our general ICU from the surgical ward where he was initially admitted with mild AHRF due to polytrauma conditioning left side rib fractures and a small contusion in the left inferior lobe. Upon ICU admission, he was diagnosed with septic shock (nosocomial pneumonia as the primary site of infection) and required invasive mechanical ventilation. During the initial period of his ICU stay, although an improvement in multiple organ dysfunction was observed, severe AHRF persisted. Moreover, O_2_ A-a grad of 30 mmHg and positive bedside contrast saline echocardiography confirmed the HPS diagnosis. In this study, we discuss the diagnostic approach of HPS and the increasing relevance of contrast saline echocardiography at the bedside, particularly in critically ill patients. The performance of this technique by trained intensivists at the bedside in the ICU minimises critical moments, such as the time required for intra-hospital transport of patients for complementary examinations, considering they have severe ventilatory compromise, thereby allowing HPS diagnosis with high sensitivity.

## Introduction

Hepatopulmonary syndrome (HPS) is a severe complication of chronic liver disease (CLD), characterised by the presence of hypoxaemia induced by intrapulmonary vascular dilatations [1-4]. Platypnea is highly suggestive of HPS given its high specificity but is reported only in 18-20% of the cases [1,3]; however, progressive dyspnoea is the most frequently reported symptom, which may camouflage the former, and thus, lead to HPS's underappreciation. Furthermore, HPS may be underdiagnosed because dyspnoea is non-specific and highly prevalent in CLD due to several other complications, such as ascites, hydrothorax and CLD-related cachexia that characterise the baseline disease of these patients [1-4]. Moreover, the diagnostic criteria are not consensual, with the reported incidence being highly variable, ranging between 4%-32% in patients with cirrhosis [1,3,4]. Regarding the imaging modalities for the identification of intrapulmonary vascular dilatations, several techniques exist, with saline contrast echocardiography (transthoracic or transoesophageal) being the most sensitive tool for a confirmed diagnosis [5,6].

## Case presentation

Case 1

A 37-year-old man with a previous medical history of arterial hypertension and alcoholic CLD with portal hypertension (PH) was initially admitted to the internal medicine ward for decompensated cirrhosis with hypoxaemic acute respiratory failure (ARF) based on large-volume hydrothorax, with an arterial blood gas (ABG) analysis at admission revealing a partial pressure of oxygen (PaO2) of 60 mmHg (reference value > 60 mmHg) and partial pressure of carbon dioxide (PaCO2) of 30 mmHg (reference value 35-45 mmHg) in room air (Table 1). The initial oxygen (O2) therapy at the ward was nasal cannulae at 4L/min of O2 flow, with oxygenation improvement.

**Table 1:** Arterial blood gas analysis of the first patient upon hospital admission revealing a mild acute hypoxemic respiratory failure with hypocapnia PaO2: partial pressure of oxygen; PaCO2: partial pressure of carbon dioxide; HCO3^−^: bicarbonate * values not in the normal range

Parameters	Results	Reference values
pH	7.37	7.35–7.45
PaO_2 _(mmHg)	60 *	>60
PaCO_2 _(mmHg)	30 *	35–45
HCO3^− ^(mEq/L)	20 *	22–26
Lactate (mmol/L)	0.8	<2

During the initial days of hospitalisation, besides pleural drainage and diuretic optimization, the patient showed progressive worsening of hypoxaemia and on day three he was evaluated by our rapid response team (RRT) for severe respiratory distress (increasing respiratory effort and utilisation of accessory muscles). The patient demonstrated severe ARF with respiratory acidosis, with an ABG analysis revealing a PaO2 of 50 mmHg and PaCO2 of 50 mmHg on inspired oxygen (FiO2) of 40% using a venturi mask. In addition to respiratory compromise, we also identified haematological dysfunction with thrombocytopenia (platelet count: 50 000/µL; reference value: 150-400000/µL), hepatic dysfunction with hyperbilirubinaemia (total bilirubin: 9.94 mg/dL), coagulopathy with an international normalised ratio of 3.34 and renal dysfunction, suggestive of hepatorenal syndrome (Table 2).

**Table 2:** Laboratory workup of the first patient upon hospital admission revealing haematological, hepatic, and renal dysfunctions * values not in the normal range

Parameters	Results	Reference values
Haemoglobin (g/dL)	7.8 *	11.8­-15.8
Leucocytes (µL)	4.30	4.0­-10.0
Platelets (10^9^/µL)	50 *	150­-400
Urea (mg/dL)	50 *	17-­43
Creatinine (mg/dL)	1.7 *	0.6-­1.0
Total Bilirubin (mg/dL)	9.94 *	0.3-­1.2
Alkaline phosphatase (IU/L)	220 *	30–120
Gamma-glutamyl transferase (IU/L)	400 *	<55
Aspartate transaminase (IU/L)	45 *	8–35
Alanine transaminase (IU/L)	99 *	10–45
C-reactive protein (mg/dL)	0.8	0.01–0.82
International normalised ratio	3.34 *	<1.5

**Figure 1:**
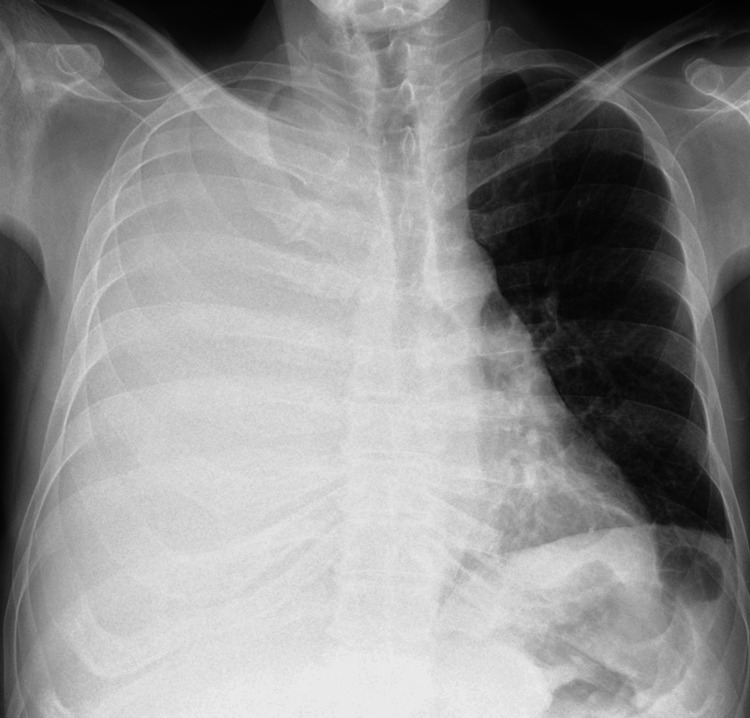
Chest radiography of the first patient revealing a large-volume right pleural effusion upon ICU admission

Subsequently, he was transferred to the ICU for further stabilisation and diagnosed with acute-on-chronic liver failure (ACLF) grade 2. Upon ICU admission, the patient underwent chest radiography revealing a right large-volume pleural effusion (Figure 1).

**Figure 2:**
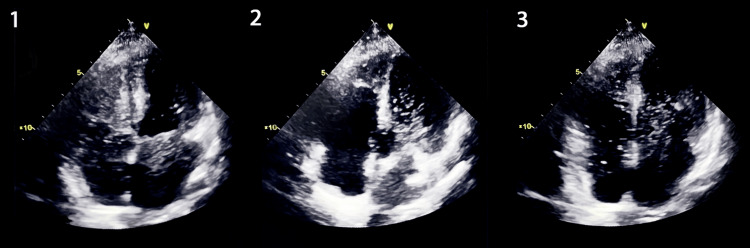
Contrast saline transthoracic echocardiography of the first patient in three phases. Sequence 1 revealing micro-bubbles in the right chambers at the moment of saline injection; sequences 2 and 3 revealing micro-bubbles in the left chambers after the sixth and seventh cardiac cycles, respectively

Consequently, he underwent pleural drainage followed by invasive mechanical ventilation. The pleural fluid analysis was compatible with a transudate (pleural fluid protein/serum protein < 0.5 and pleural fluid lactate dehydrogenase (LDH)/serum LDH < 0.6), consistent with the initial hydrothorax diagnosis. Despite its reduction with pleural drainage and a negative fluid balance in the initial ICU days, severe hypoxaemia continued to persist and on day seven we identified orthodeoxia with an improvement in PaO2/FiO2 (P/F) ratio from 126 at 35º of head of bed elevation to 164 at 0º. At this point, the alveolar-arterial oxygen gradient (O2 A-a grad) was 27 mmHg (normal value < 15 mmHg). Given the presence of orthodeoxia and the elevated O2 A-a grad, HPS was suspected. In this context, we performed a contrast saline transthoracic echocardiography at the bedside that revealed microbubbles in the cardiac left chambers six beats after peripheral injection, suggesting the presence of intrapulmonary shunt, thereby confirming the diagnosis (Figure 2).

In the following days, the patient was mainly positioned in the Trendelenburg position with a slight improvement of oxygenation with this manoeuvre. The possibility of orthotopic liver transplantation was discussed with a tertiary centre, but the patient was not considered a candidate owing to the severity of all the other dysfunctions and his baseline frailty (a baseline C Child-Pugh score and a Model for End-Stage Liver Disease (MELD) score of 28). Thus, gradual weaning of mechanical ventilation was attempted, and the patient was extubated to non-invasive ventilation on day 12 of the ICU stay and transferred to the internal medicine ward on day 14. The patient was found in cardiac arrest on day 21 at the internal medicine ward, in asystole, and a joint decision was made to withhold resuscitation efforts.

Case 2

A 58-year-old man with a medical history of alcoholic CLD with PH was admitted to the surgical ward of our hospital after polytrauma with a diagnosis of mild ARF (Table 3) (P/F ratio 286; (mild ARF range 200-300)) based on left side rib fractures (one point of fracture from the fifth to the tenth rib, with no flail chest criteria) and a small contusion in the left inferior lobe.

**Table 3:** Arterial blood gas analysis of the second patient upon hospital admission revealing mild acute hypoxemic respiratory failure PaO2: partial pressure of oxygen; PaCO2: partial pressure of carbon dioxide; HCO3^−^: bicarbonate * values not in the normal range

Parameters	Results	Reference values
pH	7.36	7.35–7.45
PaO_2 _(mmHg)	60 *	>60
PaCO_2 _(mmHg)	43	35–45
HCO_3_^− ^(mEq/L)	23	22–26
Lactate (mmol/L)	1.3	<2

**Figure 3:**
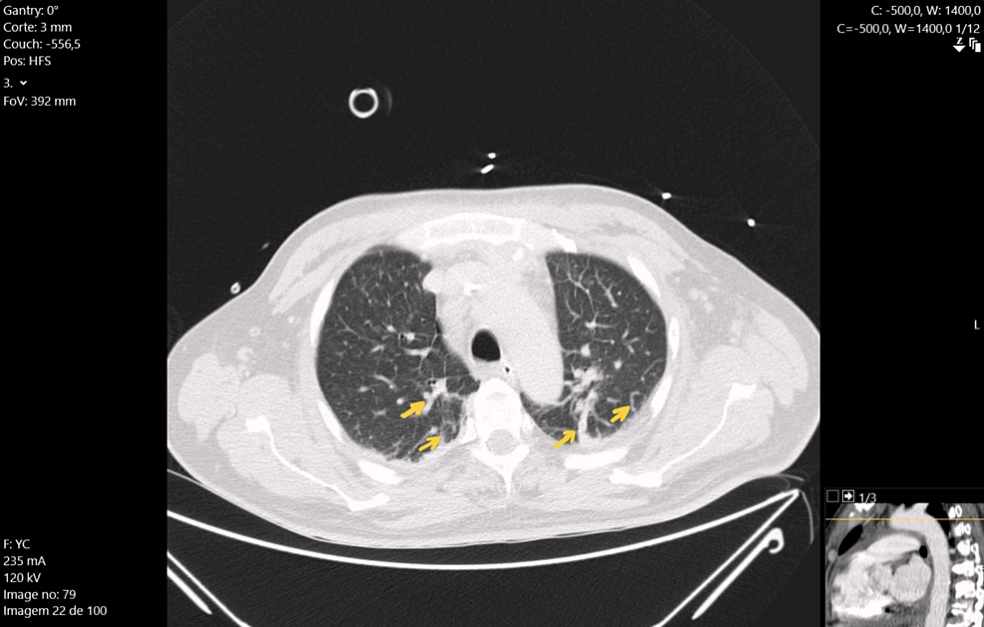
Thoracic CT scan revealing a bilateral peripheral linear image suggestive of vascular dilatations (yellow arrows)

On day four of hospitalisation, he was evaluated by our RRT for desaturation, with a SpO2 of 80%. In addition to respiratory dysfunction with respiratory acidaemia (pH of 7.32, PaO2 of 50mmHg and PaCO2 of 58 mmHg), cardiovascular dysfunction with mean arterial pressure of 62 mmHg, hyperlactacidaemia with serum lactate level of 4mmol/L (reference value < 2 mmol/L) and increased capillary refilling time was also observed. Consequently, he was admitted to the ICU with a diagnosis of septic shock with the most probable primary cause of the shock being nosocomial pneumonia, and antimicrobial therapy with piperacillin/tazobactam was initiated. Upon ICU admission, invasive mechanical ventilation and aminergic support with noradrenaline (up to 0.5 µg/kg/minute) were initiated. During the initial four days in the ICU, there was an improvement in cardiovascular and renal dysfunctions and inflammatory laboratory parameters, such as apyrexia and reduction of bronchial secretions, but severe ARF persisted, with a P/F ratio <100. Then, the patient underwent a high-resolution thoracic CT scan (Figure 3) that revealed bilateral basal non-specific peripheral linear infiltrates, suggesting intra-pulmonary shunts.

As HPS was suspected, with an O2 A-a grad of 30 mmHg, we performed a contrast saline echocardiography that confirmed the diagnosis. In the following days, the patient was preferentially positioned in the Trendelenburg position, and an off-label dietary supplementation with garlic (Allium sativum), reported to decrease nitric oxide synthesis in a few cases, was implemented [3,7]. Given the progressive improvement in respiratory dysfunction, the patient was extubated on day 16 and moved to the surgical ward on day 18. The patient was discharged on day 24 but was readmitted one month later for decompensated cirrhosis with ARF and severe hypotension; given his elevated frailty, a joint decision was made, and a do not escalate order was decided upon.

## Discussion

The diagnostic criteria for HPS are as follows: (1) CLD and/or PH; (2) hypoxaemia with a PaO2 <80 mmHg or O2 A-a grad >15 mmHg in room air, and (3) imaging documentation of pulmonary vascular dilatations [1,2,4]. Several complementary tools exist for evaluating vascular dilatations, namely contrast saline echocardiography, lung angiography, and perfusion studies with macro-aggregated albumin [1-6].

Contrast saline echocardiography is widely used owing to its low invasiveness and high sensitivity for the evaluation of qualitative vascular lesions [3,5]. Under normal conditions, the micro-bubbles created by the agitated saline do not cross into the left cardiac chambers because of their superior dimensions (> 10 μm) compared with the normal lung capillaries (8 μm). Thus, they get trapped in the pulmonary circulation and are absorbed by the pulmonary alveoli [3,5]. In the presence of pulmonary dilatations, the microbubbles can cross the pulmonary capillary bed and reach the left cardiac chambers and can be identified between the fourth and sixth cardiac cycles following peripheral vein injection. Therefore, contrast saline echocardiography is a practical and minimally invasive diagnostic method for HPS and is the gold standard for HPS diagnosis [5-7]. Transoesophageal echocardiography with the same contrast method exhibits higher sensitivity but is not preferred in this sub-group of patients owing to the frequent presence of oesophageal varices and the non-minimal risk of lesions [5].

Regarding hypoxaemia, its diagnosis through O2 A-a grad has a higher sensitivity than PaO2 alterations with body positioning (orthodeoxia). The diagnosis is confirmed when the O2 A-a grad is >15 mmHg, as previously mentioned. Furthermore, the HPS severity can be established according to the baseline PaO2 in room air with the following values: mild: PaO2 >80 mmHg; moderate: 80 mmHg > PaO2 > 60 mmHg; severe: 60 mmHg > PaO2 > 50 mmHg; very severe: PaO2 <50 mmHg [8-10].

Therapeutic approaches include off-label options directed towards the physio-pathological mechanisms of this syndrome, such as nitric oxide synthetase inhibitors, such as nebulized N(G)-nitro-L-arginine methyl ester, but orthotopic liver transplantation is the only procedure with prognostic impact. Thus, as these patients are indicated for prioritization in the transplant list, an early diagnosis is crucial for identifying this disease [1-4].

## Conclusions

In the two presented cases, the clinical suspicion of HPS was challenging owing to the initial presence of ARF relying on a different aetiology but persisting during the ICU stay despite the resolution of the initial respiratory pathological process. Transthoracic echocardiography at the bedside was essential for our patients, allowing minimisation of intra-hospital mobilisations for confirming the diagnosis. This method reduced potential complications and instability instances associated with transport and, simultaneously, optimised diagnosis timing.
